# Differential Attainment Within the Specialised Foundation Programme: Creating an Accessible Mentorship Scheme to Increase Diversity Within Academic Medicine

**DOI:** 10.7759/cureus.47700

**Published:** 2023-10-26

**Authors:** Joseph Salem, Stephen Robertson, Nadine Paul, Alokya Balagamage, Humza Awan

**Affiliations:** 1 Department of Otolaryngology, London North West University Healthcare NHS Trust, London, GBR; 2 Department of Otolaryngology, South Tyneside and Sunderland NHS Foundation Trust, Sunderland, GBR; 3 Department of Medical Education, King's College London, London, GBR; 4 Department of Metabolism, Digestion and Reproduction, Imperial College London, London, GBR

**Keywords:** mentorship programme, online teaching, diversity and equity in medicine, accessibility, differential attainment, student education

## Abstract

The Specialised Foundation Programme (SFP), formerly the Academic Foundation Programme, is a highly competitive pathway into academic medicine. There is minimal information available on the demographics of those who apply to the programme, how it scores its applicants and who is successful, making it difficult to assess whether the application process is accessible to all students and promotes a diverse workforce. There are varying levels of support available with coaching, either geographically ring-fenced by universities or available through paid courses. As a result, there is a risk of differential attainment between students who have financial constraints or attend universities where the SFP is less promoted. The aim of the study was to assess student opinion on barriers to the SFP and academic medicine and the demand for the creation of a national, free-to-access SFP mentorship programme to reduce differential attainment amongst student cohorts. Students in the programme received mentorship, peer learning and scheduled teaching events over a six-month period. Surveys were distributed pre- and post-course, and qualitative and quantitative analysis was conducted. Of the respondents, 76% felt that medical schools provided insufficient information on SFP, 31% did not feel financially stable at university and 53% stated that they would not enrol if a cost was present. Applicants were tested on pre- and post-course confidence, all of which showed an increase in mean Likert (1-5) scoring post-mentorship. Financial, institutional and geographical barriers to students applying to the programme were identified. Whilst further research is required to better understand the barriers to academic medicine, national, free-to-access mentorship may effectively reduce differential attainment and improve accessibility amongst students.

## Introduction

Differential attainment within medical education is a key indicating factor for systemic inequalities experienced by different groups of students, and it has been shown to be highly prevalent in medical education and foundation school allocation [1,2]. It can be described as the unexplained variation in achievement between two groups: one that has a protected characteristic and one that does not [3,4]. Importantly, it is the variance in average performance, not that of an individual. Protected characteristics are defined by the Equality Act 2010 and include discriminable qualities such as ethnicity, gender and disability [5].

The Specialised Foundation Programme (SFP) is the first stage after medical school where students can embark on the beginning of an academic career [6]. It is a competitive process that includes a points-based system, with marking dependent on achievements by the applicant, alongside an interview. The overall process varies depending on which Academic Unit of Application (AUoA) is applied for [7]. Due to the competition for places, there is a market for courses that provide insights and coaching from previous applicants leading to a higher chance of a successful application [8]. These are often courses that the student will have to pay for or free courses run by specific societies within certain institutions or universities that are regionally ring-fenced. There has been a concerted effort as a result to reduce these financial, institutional or geographical barriers but may still have a significant influence over which students are successful in applications to the SFP [9]. As a result, these barriers may affect which students will embark on an academic medical career.

Mentorship has been offered as an effective tool in narrowing the gap in student performance and is built into the General Medical Council's professionalism and training guidelines [10]. Of healthcare professionals, 70% believe that mentorship would increase confidence and work-study transition and improve anxiety [11], job satisfaction and motivation [12]. This is particularly important given that multiple studies show that anxiety and depression are high in medical school populations [13]. If mentorship can be delivered via an online, accessible and free resource to students applying to academic posts, it may have the potential to overcome some of the current barriers present in accessing academic medicine, increasing diversity and reducing differential attainment.

We present the outcomes of a new virtual free-to-access SFP mentorship programme that aims to increase the accessibility and diversity of applicants to the Specialised Foundation Programme.

## Materials and methods

Programme overview

The mentorship program, titled "The Academic Medic", was conducted in collaboration with King's College London starting in August 2022. This six-month online initiative offered both teaching and mentorship, accessible to medical students throughout the United Kingdom at no cost. The programme has been iteratively refined annually since 2020, incorporating student feedback with each year group.

Programme content

For the 2022 cohort, fortnightly centralised teaching sessions were conducted via the Zoom video platform (Zoom Video Communications, San Jose, CA). Each session focused on essential SFP principles such as application strategies, interview preparation, clinical scenario discussions, critical appraisal techniques and interactive mentor and mentee sessions. All teaching components were conducted by alumni who had successfully secured an SFP place, and each session spanned approximately 1.5-2 hours, inclusive of a question-and-answer segment.

Alongside the teaching component, students were offered the opportunity to be involved in a mentorship scheme where students were allocated to peer groups based on their regional deanery preferences. Groups were capped at five members to facilitate effective peer-to-peer interactions. Each group was further guided by a dedicated academic mentor with first-hand experience in successfully completing the SFP in their respective area. Communication within mentor groups was facilitated via WhatsApp, and students were encouraged to convene their own peer discussions in addition to bi-weekly mentor-led meetings on Zoom. Topics for these meetings were tailored to the educational needs of each group. Mentorship engagement was monitored via monthly mentor feedback.

Student recruitment

Information about the programme was disseminated through various channels including social media platforms (Facebook, Instagram and Twitter) specifically targeting medical school societies, as well as through direct emails to society representatives and education leads in medical schools across the UK. Eligibility criteria specified that applicants must be in their final year of medical school and applying for the Specialised Foundation Programme (SFP) in the current academic year. Students applying to the Specialised Foundation Programme only or not in their final year of medical school were not enrolled on the course. All students who fit the eligibility criteria were accepted onto the course with no limitations on the number of places available.

Mentor recruitment

Successful alumni of the course who had secured SFP places were recruited as mentors for the 2022 cohort. The eligibility criteria for mentors were that they were current SFP doctors and could commit to mentoring up to five students for the whole duration of the course. Mentors were given advice on coaching techniques and topics before the course started and were able to contact the course organisers at any time during the course to discuss questions or issues. Mentor feedback was collected at the end of the mentorship programme using Google Forms (Google, Inc., Mountain View, CA).

Data collection and analysis

Prior to initiating the mentorship and teaching segments, accepted students were required to complete a pre-programme questionnaire using Google Forms. This survey was mandatory and incorporated a range of question types including white space, multiple choice and Likert scale questions. These aimed to capture student demographics, educational requirements and prior SFP exposure and preparation. Students who failed to complete this initial survey were precluded from programme participation.

Feedback was systematically collected at the beginning and conclusion of each teaching and mentorship session via Google Forms. These surveys consisted of a combination of closed- and open-ended questions, enabling both quantitative and qualitative data analysis. At the end of the mentorship period, participant interviews were conducted to better understand how the course could improve with the qualitative information collated. Data was collated and analysed using Microsoft Excel (version 16.77.1) (Microsoft Corp., Redmond, WA), with graphical representation performed through GraphPad Prism (version 10.0.3) (GraphPad Software Inc., La Jolla, CA) and thematic analysis performed on qualitative data.

## Results

The programme was delivered on a yearly basis (2020/2021/2022) from August to January. Given the iterative improvements made each year the scheme ran, the data presented is from our 2023 cohort, unless otherwise stated.

In our 2023 cohort, we received 93 responses to our initial participant survey. Of the participants, 79% were female, and 93% were between the ages of 21 and 25 years. A mix of ethnicities was included, as represented in Table 1. Of the participants, 75% were intercalated, and 17% were completing medicine as a postgraduate. Of the students, 10% were international. Students were based at 11 different medical schools including Barts University, Queen Mary University, King's College London, Norwich University, Dundee University, Cambridge University, Nottingham University, Oxford University, Bristol University, Birmingham University and University College London.

**Table 1:** Demographics of students applying for "The Academic Medic" SFP mentorship course SFP: Specialised Foundation Programme

Age	
21-25 years	93%
25-30 years	7%
Gender	
Male	21%
Female	79%
Degree	
Undergraduate	83%
Postgraduate	17%
Self-reported ethnicity	
White British	14%
White non-British	14%
Asian	39%
Arab	7%
Black	7%
Mixed	7%
Preferred not to disclose	12%

Prior to university, 50% of the students reported going to a non-fee-paying school, whereas 33% reported attending a fee-paying education. The remainder preferred not to disclose this information. Regarding barriers to course access, 69% of the students felt financially stable whilst at university, yet 69% had not enrolled on a specific course they felt would benefit their grades and medical knowledge because of cost. Of the students, 41% felt that "free-to-access" courses were of the same quality in content and delivery as paid courses. When questioned on how much they would pay for a teaching and mentorship programme during their SFP application process, 53% stated that they would not enrol if a cost was present; however, 39% suggested that they would pay up to £100, and 9% may pay up to £300.

**Figure 1:**
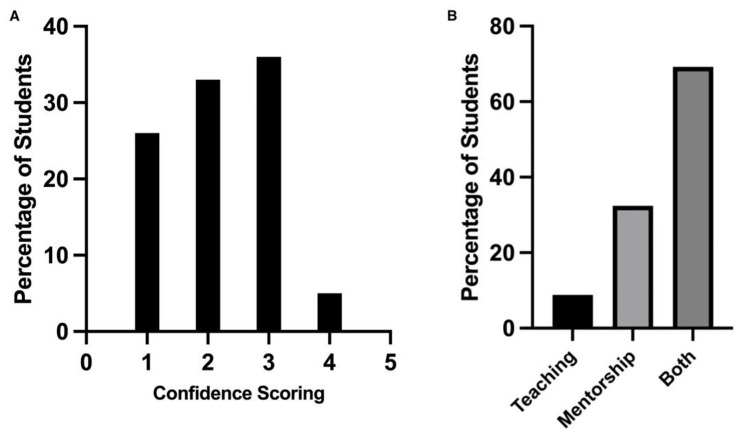
(A) Graph showing student confidence in securing an SFP position (1-5 Likert scale) and (B) graph showing the perceived "most valuable" function of the mentorship programme SFP: Specialised Foundation Programme

**Figure 2:**
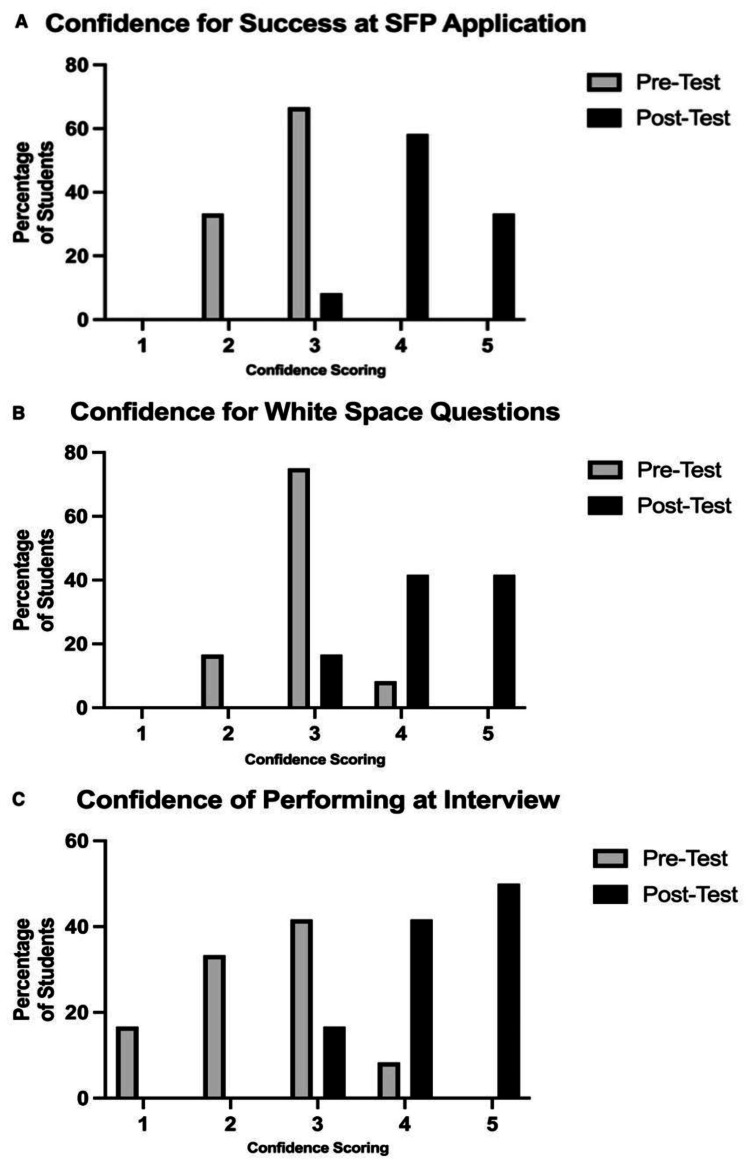
Pre- and post-course student confidence scoring: (A) perceived confidence in a successful SFP application, (B) perceived confidence at forming high-quality white space questions and (C) perceived confidence at performing well at interview SFP: Specialised Foundation Programme

Regarding where students first heard about the SFP, 63% were provided with information from their university, 24% from word of mouth and 13% from social media or external talks. Of the respondents, 76% felt that medical schools provided insufficient information on SFP. Student confidence regarding the successful attainment of an SFP place was also assessed (graded using a Likert scale). This is represented in Figure 1, with 1 rated as not confident at all and 5 indicating that they were very confident: 26% scored a 1, 33% a 2, 36% 3 and 5.4% a 4. No participant scored a 5. In terms of educational needs, 8.8% felt that a teaching series on the SFP would be most beneficial, whereas 32.4% wanted mentorship only. The majority, 69.2%, believed that both teaching and mentorship were necessary for their success as demonstrated in Figure 2.

Students were questioned on their reasoning for applying to SFP. The most common answers included "interest in research", "having previously had a dedicated research project/selected component", "family support/from a medical or academic family" and "support from the university regarding SFP applications". Conversely, reasons for not applying for the SFP included "lack of SFP application support from the university", "lack of access to someone previously completing SFP", "lack of awareness regarding the SFP" or "the belief they do not have enough research experience/strong enough application".

The post-course questionnaire received a 30% response rate. Greater than 97% rated the teaching event as good or very good, and 94.9% rated it as useful or extremely useful. For these students, we assessed their pre- and post-course confidence relative to completing the application successfully, for the white space questions and for performing at interview. As represented in Figure 2, there was a notable improvement in confidence across all tests, with an increase in mean scores of 1.58, 1.33 and 1.91, respectively. Overall, 91% of the 40 mentored students in our 2022-2023 cohort received successful SFP offers.

## Discussion

The most up-to-date release of the United Kingdom Foundation Programme Office (UKFPO) recruitment statistics show that in 2021, the AUoA had a national average of 4.54 applicants per advertised post [14]. This ranged as high as 9.90 per job in some regions. It is a highly competitive programme, and whilst no data is available to show the prevalence of differential attainment on applications to this specific programme, it can be extrapolated that those with protected characteristics that are at a disadvantage during medical school and standard foundation programme applications are also at a disadvantage during applications to the SFP [2,4]. Despite this high competition ratio, 91% of mentored students received successful SFP offers, showing the potential beneficial impact of this mentoring programme. It must be considered that the cohort of students, all of whom applied through either social media advertisements or emails from their medical school, may well represent a particularly motivated group of individuals. Nonetheless, a 91% success rate is significantly higher than the 22% national average. Seeing as there was a recordable increase in average confidence level after the course and its included mentorship, we hope that these results will be reproducible on further testing.

Whilst this course was provided with no charge, 53% of the students stated that they would not have been willing to pay for this support. However, when combined with the highly successful SFP offer rate of 91%, it demonstrates the effect that courses can have on attainment levels. Financial constraints have a risk of disproportionately affecting those from minority groups, such as those with protected characteristics [15], and this may show the possible effect paid courses have on differential attainment. Therefore, the removal of financial barriers, where possible, may improve equality in accessing mentorship and training for the SFP.

Another barrier to application to the SFP was the apparent lack of exposure to research as a medical student. Exposure is often dependent on the university syllabus, student selective components or undertaking intercalated degrees. Intercalation can serve as a platform to experience academia and is thought to improve overall undergraduate results, yet it comes with a significant monetary cost [16]. Whilst the NHS bursary can cover some of the costs, it does not account for all the increase in debt from living expenses and the delay and/or loss of a year's earnings over your career [17]. With different universities offering different research experiences, universities having different research funding models [18] and some universities having closer relationships with innovation and research hubs [19], university choice and therefore geographical location may also effect.

University support was shown to be highly important in influencing student application and may have a pivotal role in student choice. Students who received university support were more confident in their applications, with the absence of support adversely impacting students and their likelihood to apply for the SFP. This should be of significant concern to universities that do not appropriately support students, especially those with protected characteristics, and this could be modified to help reduce differential attainment with targeted support for these students. This could include explaining the potential career benefits of research placements and providing financial support for students that require it, increasing SFP exposure through talks and inviting current SFP doctors to talk about the application process and their experiences alongside offering SFP mentorship programmes.

## Conclusions

There are a number of financial, institutional and geographical barriers to students applying to the programme. This may limit how accessible the SFP is to medical students. More research is needed to understand the effect of these barriers on diversity and differential attainment alongside better planning and provisions to further improve accessibility. Free-to-access mentorship may effectively reduce differential attainment irrespective of student background.

Having a scalable and effective, centrally controlled, but regionally delivered mentorship scheme may create effective mentorship partnerships. Having a formalised national free-to-access SFP mentorship scheme available to all at the time of application could help equalise the inter-university variation in SFP support, increase awareness of the SFP and improve individual confidence, especially in students who have not had access to dedicated research opportunities or exposure to successful SFP applicants.
